# “To be seen” – older adults and their relatives’ care experiences given by a geriatric mobile team (GerMoT)

**DOI:** 10.1186/s12877-021-02587-y

**Published:** 2021-11-06

**Authors:** Iréne Ericsson, Anne W. Ekdahl, Ingrid Hellström

**Affiliations:** 1 School of Health Sciences, Jönköping University, 553 18 Jönköping, Sweden; 2 Department of Clinical Sciences Helsingborg, Helsingborg Hospital, Lund University, Almahuset, Svartbrödregränden 3–5, Fack 2, 252 23 Helsingborg, Sweden; 3 The Department of Health Care Sciences, Palliative Research Centre, Ersta Sköndal Bräcke University College, 116 28 Stockholm, Sweden

**Keywords:** Qualitative study, Multimorbidity, Comprehensive geriatric assessment, Care experience, Community dwelling, Safety, Security

## Abstract

**Background:**

The proportion of older people in the population has increased globally and has thus become a challenge in health and social care. There is good evidence that care based on comprehensive geriatric assessment (CGA) is superior to the usual care found in acute hospital settings; however, the evidence is scarcer in community-dwelling older people.

This study is a secondary outcome of a randomized controlled trial of community-dwelling older people in which the intervention group (IG) received CGA-based care by a geriatric mobile geriatric team (GerMoT). The aim of this study is to obtain a better understanding, from the patients’ perspective, the experience of being a part of the IG for both the participants and their relatives.

**Methods:**

Qualitative semistructured interviews of twenty-two community dwelling participants and eleven of their relatives were conducted using content analysis for interpretation.

**Results:**

The main finding expressed by the participants and their relatives was in the form of feelings related to safety and security and being recognized. The participants found the care easily accessible, and that contacts could be taken according to needs by health care professionals who knew them. This is in accordance with person-centred care as recommended by the World Health Organisation (WHO) for older people in need of integrated care. Other positive aspects were recurrent health examinations and being given the time needed when seeking health care. Not all participants were positive as some found the information about the intervention to be unclear especially regarding whom to contact when in different situations.

**Conclusions:**

CGA-based care of community-dwelling older people shows promising results as the participants in GerMoT found the care was giving a feeling of security and safety. They found the care easily accessible and that it was provided by health care professionals who knew them as a person and knew their health care problems. They found this to be in contrast to the usual care provided, but GerMoT care did not fulfill some people’s expectations.

## Background

The proportion of older people in the population continues to increase globally, which is causing challenges in regard to health and social care [1]. Among people 80 years old or older, over 80% have two or more diagnoses of chronic diseases, and 54% of people over 85 years old have four or more such diagnoses [2, 3]. One of the reasons for the increased incidence of multimorbidity in the population is improved medical care and the development of treatment options for chronic diseases. Therefore, the fact that more people are surviving with multimorbidity is a sign of success in health and social care; however, as a consequence, more people are living with reduced functional ability, which has a negative impact on the older person’s well-being and quality of life in the last years of their existence [4, 5]. Older people with multimorbidity frequently need to seek help in health care, and there is a connection between the number of concurrent diagnoses and health care costs [6, 7]. The Swedish Agency for Health Technology Assessment and Assessment of Social Services (SBU) reviewed interventions with the aim of improving care for older people with multimorbidity who are acutely admitted to hospitals. The results showed that older people with multimorbidity constituted 40% of all emergency visits, and the only intervention that significantly improved their care was the Comprehensive Geriatric Assessment (CGA) [1]. The CGA is defined as a multidisciplinary diagnostic and treatment process that identifies the medical, psychosocial, and functional limitations of a frail older person to develop a coordinated plan to maximize their overall health with ageing [8, 9]. This care plan is updated when needed by a team, which at a minimum consists of a physician, nurse, and occupational and physiotherapists but often has support from, for instance, dieticians, social workers, and psychologists.

The latest Cochrane report on the effect of the CGA from 2017 concluded that there was a high level of evidence that the CGA increases the likelihood that patients will be alive and living in their own homes at the 3- to 12-month follow-up after an acute in-hospital care period. The same report concluded that the likelihood that patients would be admitted to a nursing home at the 3- to 12-month follow-up decreased [9]. A study on patients cared for in an orthogeriatric ward compared to an orthopaedic ward showed that the orthogeriatric ward (with care based on the CGA) proved to be cost-effective and to positively affect the person’s functional ability compared to the orthopaedic ward [10]. Therefore, CGA-based care is beneficial for older patients. Despite this, most hospital health care is not organized in this way but is rather based on different medical specialties. However, what about the care of community-dwelling older people? According to the WHO, older persons with multimorbidity need integrated care with a holistic view, and a focus on multiple diagnoses is required [1, 11, 12].

A national survey (NS) consisting of qualitative interviews with older people with multimorbidity, which was performed by The Swedish Authorities of Health and Social Care (SKR), showed extensive health- and social-care shortcomings [13, 14]. All the participants were identified via local health care registries with the following inclusion criteria: community-dwelling, age ≥ 75 years, ≥ 3 different diagnoses according to different chapters in the ICD-10, and ≥ 3 in-hospital care periods during the past 12 months [13]. In this way, older people with a high level of health care consumption were defined, as this population accounts for 19% of all the costs of in-hospital care in Sweden [15]. The NS included 298 older people and 265 relatives from 12 different regions of Sweden [14], and the outcomes were the same all over the country. The coordination and transfer of information between different caregivers had apparent weaknesses, there was a lack of continuity of care, and it was seldom that any caregiver took full responsibility for all the medical treatments of a patient. In addition, the survey showed that relatives, in their opinion, were not sufficiently involved in how care was designed in regard to health or social care [13, 14]. Relatives account for approximately two-thirds of all the caregiving of older people with multimorbidity in Sweden; this number has been shown to be stable over time [16, 17]. Overall, the survey showed a fragmented health care system for older people with multimorbidity with a lack of continuity despite the very high costs for health and social care. There have been later evaluations of the role of primary care (PC), but PC still suffers from a lack of continuity, and no overall improvement has been seen in the last 10 years [18].

During 2011–2013, a randomized controlled trial was conducted, namely, the Ambulatory Geriatric-Frailty Intervention Trial (Age-FIT) [19], which included 382 community-dwelling older persons with multimorbidity. The inclusion criteria were similar to that used in the NS, namely, community-dwelling individuals aged ≥75 years who had received inpatient hospital care three or more times in the previous 12 months and had three or more concomitant medical diagnoses. For 208 of the 382 participants, the intervention consisted of access to a geriatric team that provided CGA-based care as a supplement to the usual care. The control group had access to the usual care, which in Sweden means PC, community services, and in- and outpatient hospital care. Usually, most care is given when the patients seek it; the health care system rarely provides prescheduled proactive health visits. Both the intervention and control groups had access to PC, hospitals, and ambulatories under equal conditions. Normally, usual care is not organized as care given by a team, and with regular team meetings, the patient will seek one professional at a time, e.g., physician/nurse/physiotherapist. In the AGe-FIT study, the patients were followed for 24 months after randomization. In the intervention group (IG), common measures performed by the geriatric team consisted of, e.g., changes in medication, exercise programs, referrals for investigation when health problems occurred to detect diseases, dietary supplements, or increased home help care. The evaluation of the intervention revealed results such as significantly fewer hospital days, better survival, increased sense of security, and a reduced progression of frailty for those who had been part of the intervention group [19, 20]; however, the positive results have not yet been followed up from the perspective of older persons, and the qualitative evaluation of CGA-based care in community-dwelling older persons has been sparse up till now [1, 21].

To follow up on the positive results of the AGe-FIT trial, a new randomized controlled trial, namely, the Geriatric Mobile Team study (GerMoT), has been conducted in another area of Sweden. The inclusion criteria were very similar to those of the NS and AGe-FIT studies, i.e., participants were identified through local health care registries with the following inclusion criteria: age ≥ 75 years, ≥ 3 different diagnoses and ≥ 3 visits to the emergency care unit (with or without admittance to hospital) during the past 18 months. The study protocol is described in detail elsewhere [22] and is registered at ClinicalTrials.gov ID: NCT02923843.

The present paper focuses on the perspectives of the participants who received the CGA-based intervention and their relatives, which were obtained through qualitative interviews. The reason why we chose to interview only the intervention group was due to the results already achieved in the larger NS and in other sources where later evaluations have shown no major changes in health care for older people. Additionally, it turned out that the participants spontaneously compared the care given by the GerMoT with the usual care, which is described in the findings. This gives us reason to believe that we already understand the experiences of older people who are given care as usual; thus, we now want to explore the experiences of older people given CGA-based care by a mobile team.

## Methods

### Aim

To study the experiences of care of older people with multimorbidity when care is given by a mobile team based on the CGA.

### Setting

Community-dwelling older people living in Sweden. The study was conducted in the catchment area of a medium sized teaching hospital in the south of Sweden with approximately 140,000 inhabitants.

### Design

Semistructured qualitative interviews [23] were conducted with older people who were part of the intervention group and their relatives. The interview material was analysed in accordance with inductive qualitative content analysis as described by Graneheim & Lundman [24, 25]. Ethical approval was obtained (Regional Ethics Committee in Lund, Sweden Dnr.2018: 732). The research was conducted according to standards set by the World Medical Association’s Declaration of Helsinki, and the study was guided by the principles of good clinical practice [26].

### Participants

We chose every 10th participant in the intervention group who had a value of > 20 on the cognition test, namely, the Montreal Cognitive Assessment, MoCA [27]. The respondents consisted of older people both of those who had frequent and those who had sporadic contact with the GMT during the intervention. The researcher who performed the interviews was given a list of 27 older people who met the inclusion criteria. An information letter with a request for participation was sent out, and a few days later, the prospective participants received a telephone call in which the researcher requested oral consent for participation. Some prospective participants (*n* = 5) declined participation. After obtaining oral consent, an appointment was made with the patients for an interview. In the cases (*n* = 7) where the older person lived with a close relative, the relative was asked during the telephone conversation whether they could also be interviewed. At the time of the interview, the participants were asked if there were any close relatives who would be suitable to interview and whom the researcher could contact. A special information letter addressed to close relatives with a request to participate in an interview was sent to these close relatives. All the interviewed close relatives gave their verbal consent to participate in a subsequent telephone conversation.

The older people interviewed consisted of ten men and 12 women. The close relatives consisted of seven (*n* = 7) spouses (three men and four women), along with four (*N* = 4) other interviewees who belonged to the children and grandchildren generations. A total of 33 interviews were conducted for the study; please see Tables 1 and 2 for a description of the participants in this study.

**Table 1:** Demographics of participants in the GerMoT study

Number of participants	Age (range)	Age (Average)	Age (median)	% women
22	76–97	82,4	82	45

**Table 2:** Characteristics of close relatives interviewed

Number of participants	Kind of relation	% women
11		73
7	living with the participant	55
4	Younger generation	100

### Procedure

The data collection took place during February and March 2019. The researcher who conducted the interviews was from another area of the county and was thus unknown to the participants and had no knowledge of the people involved. The interviews with the participants were conducted in the older person’s home after written consent was given. The interviews with relatives were conducted either in the relative’s home (*n* = 5) or via recorded telephone calls (*n* = 6). A semistructured interview guide [23] was used when interviewing the participants and the relatives. The focus of the questions was on experiences underwent when the patients received care from a geriatric team. In some cases, the close relative was present in the room during the interview with the older person based on the person’s wishes. Therefore, these interviews were counted as both an interview with the older person and with their relative, as both individuals answered the asked questions. The interviews were recorded on a digital voice recorder, and the time of the interviews varied between 10 and 27 min for study participants and between 7 and 25 min for the relatives.

### Data analysis

The verbatim printed interview material was analysed inductively with a focus on the manifest content [25]. The analysis was carried out in collaboration between the authors, and there was a constant discussion about the findings. All the interviews were read repeatedly by IE and IH to get a feel and understanding of the whole.

Subsequently, the so-called meaning units [25] in the interview material were identified. The focus of the interview was on the participants’ experiences of the intervention, i.e., receiving care from a geriatric team in addition to regular care. In the next step of the analysis, the meaning units were condensed, i.e., the meaningful parts of the text were described in fewer words but without losing the original meaning [25]. Each condensed meaning unit was then labelled with a code that explained what the text was about. The coding was done to make the material clearer [25]. The codes were compared and contrasted, which enabled a grouping of the codes based on what codes were considered to belong together.

A comparison of similarities and differences resulted in the material being described under two themes, with some subthemes present under each theme.

### Findings

#### Perceived health and need for support

All the participants interviewed were asked about their health. Four participants (*n* = 4/22) described their health as good; one of them did so despite receiving support from the municipality. Many of the older participants (*n* = 18/22) reported that their health status varied; however, at the moment of the interviews, there was no ongoing severe illnesses reported. Of those who perceived themselves as having poor health, six (*n* = 6/18) reported that they recently had been going through a turbulent time with sickness and emergency visits and that their health might worsen rapidly.

Seven (*n* = 7/18) of the participants who received formal support or care from the municipality and/or PC perceived themselves as having poor health. The remaining eleven persons (*n* = 11/18) did not have any ongoing formal support from the municipality or PC. However, there were two participants who received help from their partners in activities related to daily life (*n* = 2/18).

**Fig. 1:**
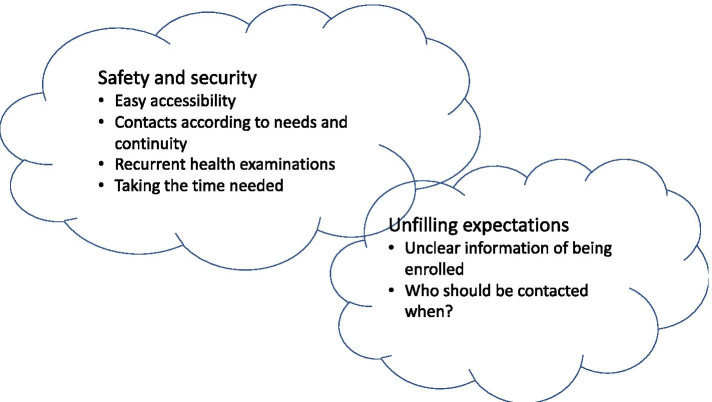
Main Themes

The analysis of the 33 interviews showed two main themes on how older persons and their relatives experienced being part of the intervention of the GerMoT (Fig. 1). One theme was “safety and security”, with the four subthemes of “easy accessibility”, “contacts according to needs”, “recurrent health examinations” and “taking the time needed”. The other main theme was “not fulfilling expectations”, with the subthemes of “unclear information” and “who should be to contacted and when?”

#### Safety and security

The main experience reported was almost exclusively benefitting from enrolment in the intervention group. The repeated contacts meant feelings of security and safety, that the older person was recognized and that someone cared for and asked about the older person.

One of the reasons for feeling secure was the possession of a direct telephone number. This meant that there was always someone to call to discuss issues or get advice in regard to some matter, which felt very valuable. Even if the need for contact did not exist for a moment, it was a safety mechanism that was in place if there should be a deterioration, which the participants knew could happen quickly. Several relatives mentioned that the participants had become more secure after joining the team, and they were happy that they also had the opportunity to call the team, which felt safe.

#### Easy accessibility

Being enrolled in the GerMoT was described by both the participants and their relatives as having easily accessible health care. The GerMoT helped with other health care contacts or planned treatments, and enrolment in the project allowed advice to be obtained regarding questions related to their health. The participants described how they often received an answer immediately when they called or that they could leave a message on the answering machine if a nurse was not available. One participating older person (27) who described having on-going ill health and no formal support or care in daily life said:

*First, I think you get in touch immediately … and if they have time … they give you time or they explain how you should do … and then when you get there, they have time for you. So, I feel now, at least when I was there last … I felt … that I received more time and more help in one hour than I probably received in one year [earlier] …* (27).

When there was a need to make contact with health care, regardless of whether the GerMoT initiated it, the participants felt as if they had a ‘VIP lane’ in the health care system. They did not have to wait in an emergency room for hours. The fact that they had a ‘VIP lane’ was even questioned in relation to fairness. They described that when visiting an emergency unit, during the time of their enrolment with the GerMoT, everything seemed to go quicker. They suggested that this ought to be a special “track” for older people at the emergency department to gain access to geriatric competence.

As one relative described:

*We have called and there has been an answering machine at some point; then, they called back as soon as they could, and she did what she should* (4).

Participant number 18, who had reported having ongoing ill health and no formal support, described the value of easy care contact:

… .*I think that when you reach the age that I am and the illnesses creeps up on you, you want … .you want … .the great value lies in the fact that you have a phone number that connects you to a person you have talked to before … because we who are in these small primary care centres, we are tired of people being replaced all the time … there are doctors and nurses … .but you do not get this contact with your “channel” in the system … and it sometimes works very poorly … So, it feels very good to have this* (18).

Although the participants described that they were aware that they should turn to PC in the first place, the experiences described above resulted in them calling the GerMoT instead. The participants and their relatives expressed that they would again consent to the participate in the project if they were asked to take part in such a study again and that they saw no disadvantages of being connected.

The participants compared the PC with the GerMoT. An older person could not call primary care just for advice; rather, an appointment had to be booked in advance. As one participant with ongoing ill health (7) stated:

*So, you have somewhere to call... you cannot call the PC and say ‘Oh, oh I feel so bad’ … you cannot do that … . then you have to book an appointment with a doctor … No, I actually think it is good* (meaning the GerMoT) (7).

The need for contact with the GerMoT was sometimes a result of the long waiting times for an appointment with the PC or that the older person’s request for an appointment had been turned down. This created a lack of trust in PC and in favour of the GerMoT.

#### Contacts according to needs and continuity

To be enrolled in the treatment group meant follow-ups regarding health status, which were conducted via telephone calls, home visits and appointments at the geriatric clinic. The older persons said that since a lot happens in regard to health during ageing, it was good that they was regularly asked about their health. There were doubts about how often the follow-up telephone calls from the team were made, but the perception was that they were done with continuity. The overall experience was that the follow-ups took place more often during periods of poorer health and then gradually returned to fewer follow-ups when one’s health was improved. One participant (29) with ongoing ill health stressed the importance of the follow-ups:

*… They call every fortnight and check how I am … Yes, now when it has been stable for a while; before, they called every week* … (29).

Some of the participants wished that the telephone follow-ups would be somewhat more frequent; however, at the same time, they concluded that it was a question of resources. Others argued that the frequency of the follow-up telephone calls was sufficient as their health was stable.

The participants described that the GerMoT retained the whole picture of the person, which meant that the person did not have to give the same information about their medical history over and over again. When the older person called the telephone number they had been given, they could talk to staff they had talked to before and who knew the person. There was a slight turnover of staff during the project; however, the older person still felt recognized. Something that was also put forward as a positive aspect was that different professions that were part of the team promoted holistic care. Several participants compared this format with that of PC, in which they described as having a major shortage in terms of staff continuity.

The experience was that the staff who took the phone calls were very competent and asked relevant questions that revealed unseen problems that could be alleviated. Alternatively, as one older person (22) described, you sometimes need help describing possible symptoms:

… *And so maybe there are problems that you do not think about … .and … when you sit and talk, they come out a little* (22).

Advice or actions initiated by the team were always followed up with a telephone call.

These follow-ups were considered even more critical for those who could not speak for themselves or did not have a close relative present to help them. The older persons and the relatives believed that these follow-ups could be seen as preventive care that might avoid emergency visits:

*Because it is better that they do them while she's healthy too … they should not only be done when one is feeling ill … … health care is a lot better if it is preventive* (4 relative).

For some of the participants, these follow-ups were the only occasions when they contacted the team. Often, they had well-functioned and ongoing care contacts (for example, private care or geriatric care nurses at PC) and did not understand why they would benefit from calling the GerMoT in case of problems. However, they were aware that the opportunity existed. Those who only had sporadic contact with the GerMoT still felt safe having the telephone number to call if the need arose. However, they expressed that it was easy to forget the possibility as contact was infrequent but that the follow-up telephone calls sometimes reminded them:

*… It (sporadic contact) … is probably the only thing that can be a little negative because you can forget that you have that opportunity* (6).

In line with this, relatives described that the older person might have wished for more regular contact with the team, but they thought that one reason for this lack of contact was that the older person did not want to disturb the team or make any fuss.

Something that was appreciated was that the examinations could be done at home if the person had difficulty travelling to the clinic. Some persons put forward that it was better to get a home visit because it was sometimes difficult to gather one’s thoughts during a phone call. Relatives also talked about obtaining a holistic picture of the person when they are examined in their ordinary environment at home.

#### Recurrent health examinations

The thorough health examinations that were performed during the visits by the nurse and the physician in connection with enrolment in the GerMoT were considered very positive.

The older persons and their relatives thought that the geriatric team provided what was described as “care”, which they have never experienced before. Several participants stated that the evaluation of their health that was done and examined so properly was very valuable precisely because they were older. It felt like a consolation that someone cared about older people. One person described the tests as ‘car maintenance’ (“thousand-mile service”).

One examination that was explicitly mentioned consisted of the cognitive tests that were done. The reason why the older persons were particularly in favour of that test was that they had a feeling that it was challenging to detect cognitive impairments themselves. Participant number two, who experienced ongoing ill health and needed assistance in everyday life, stated the importance of confirming that the “mind” was still intact:

*… I like that they test my memory and stuff … so I get to know a little about if it has gotten worse or if it has gotten better. Yes, my fear is that I should lose my mind yes … that I should not be able to cope like that* (2).

Several participants felt that they had received valuable confirmation that everything was working fine when they conducted these tests. They expressed pride over their maintained ability, although there was an awareness that this situation could change quickly.

#### Taking the time needed

Something that both relatives and the older person experienced was that there was a difference in the encounter when contacting the GerMoT versus when contacting the PC. The GerMoT was compliant and helpful, listened and let the encounter take time, facilitated other care contacts, and speeded up processes related to care and making contact with the municipality. The older persons’ experiences were that visits to their usual health care are often at a fixed day and time. This fact sometimes makes it hard for people living with multimorbidity, who need time to describe their symptoms and worries. This situation might lead to the consequence that only the most prevalent symptom is mentioned, which does not give an overall picture of the person’s situation. One relative used the expression “the clock is ticking” in regard to the usual health care services.

*… Otherwise … the clock is ticking … oh … .I have 20 minutes … what should I catch up with … what do I want to talk about … no, I have to talk about the most important …* (33).

The participants said that the accessibility and commitment present in the GerMoT is the opposite of the ‘clock is ticking’ feeling. The older persons talked about occasions in which they had been in contact with the GerMoT in emergency situations and how they then received a quick home visit. Their contact could also have been about guidance or advice about, for example, calling an ambulance and helping to receive care when needed. The comparison was made by several participants between this situation and how the PCs are organized, i.e., where a first contact is eventually made and an appointment might be delayed for days. Something that was also mentioned as a positive side of the GerMoT was that after a hospital stay or after an emergency visit, the team called them for a check-up, as a participant described:

*… And then when I came home again … the day after … … the team nurse called... and I thought it was very nice … and then I did know that there was someone who was keeping in touch with me....and then she called again after one or two day and asked how I was …* (24).

#### Unfulfilled expectations

The information provided during enrolment and in connection to the project was that participation in the GerMoT would be in addition to the usual care that was experienced by participants. Some participants wondered what the GerMoT could accomplish that their usual emergency care could not. Some explained that they would not participate if given the offer again because their expectations were not met. Disappointment was expressed by both relatives and older persons, and some areas were excluded, for example, help with mobility services, as one relative expressed in the quotation below:

*I do not truly understand this … .the purpose is to support joint efforts for older persons in addition to the usual care... with what as a supplement … I have understood that …* (mobility service), *I thought that maybe was something you could help with* … (17).

The written information about the project referred to “collective efforts,” which by some was interpreted as holistic care without omitting any parts. The anticipation was that when the person agreed to participate in the project, the team would be able to give ‘tips and tricks’ about support related to the person’s health, which was not understood to be the case. Some expressed that they did not notice any differences after joining the team apart from the follow-ups that were done in the project. Thus, the enrolment had not lived up to their expectations.

Some of the relatives were unsure if they could contact the team on behalf of the older person and thought that the older person should get in touch themselves.

#### Unclear information about being enrolled

When older people received written information about the suggestion to join the GerMoT, the reason to do so and the purpose were not entirely clear to everyone. There was a disparity among the participants regarding the anticipation of enrolment and what it could mean, which ranged from a lack of anticipation to the experience of being frustrated that it turned out to be something completely different. Some of the participants explained the sampling procedure and what they could expect when part of the intervention group. Others were still puzzled about their participation since they, in that moment, experienced good health. Nevertheless, most participants recapitulated the fact that they were asked to take part in relation to earlier periods of ill health, during which they sought emergency care frequently, and the fact that they live with multimorbidity, or with numerous conditions as one participant explained:

*… . it was probably that I have so-called multimorbidity … . I have several diagnoses …* (6).

#### Who should be contacted when?

Some uncertainty was experienced about when or on what occasions the PC should be contacted and when to contact the GerMoT. Some older people were puzzled how it came to that they had been asked to take part in the intervention in the first place. The relatives stressed that they needed more information about the GerMoT and were concerned about when the older person was asked to take part and eventually enrolled in the study. They deemed this information helpful in the event of the acute need for support or care:

*Now she was quite confused the last time she went in … . there have been other times when she went to the hospital … .but I never understood how we could use them* (the team) *… I became mostly a little annoyed with her because she was kind of stubborn that they had taken blood samples and they would at least call... if I had known that I could access this team myself, maybe we could have taken her that way* (31 relative).

However, the relatives explained that they were aware of the secrecy and the self-determination of the older people.

Despite doubts and hesitations about the reason and purpose of participation in the study, the participants gave their consent to being enrolled with the team. Reasons why they agreed to take part were curiosity and an interest in how the project would end up. There were also experiences of being persuaded to participate. Nevertheless, at the same time, the overall opinion was that by participating, you could contribute to increasing knowledge about the care of older people, which seemed to be a good thing. Some participants thought that it all seemed interesting because specialists in the care of older people were part of the team, which they felt increased the probability that, if such help were needed, more appropriate care would result if something were to happen. It was also perceived as self-evident to take part because doing so could be of importance and benefit to someone, which was seen as an exchange for the previously received care, as one participant (21) who, for the moment, was experiencing good health explained:

*… What the hell … they have taken care of my head now for 15 years … ..so, I can give back with this...that is … .if it can be of any joy for someone …* (21)

The older person did not always inform their relatives about their contact with the team. The relatives thought the reason for this was that the older people did not want to burden their relatives but also because they had a desire to manage independently. Although some participants explained that they had no expectations when they agreed to join the GerMoT, they still expressed satisfaction that they had agreed to join in. One relative stated:

… *incredibly good... that is what we can call the health care …* (8 relative)

`However, some explained that they would not choose to participate if given the offer again because their expectations had not been met.

## Discussion

The results showed that satisfaction with the intervention was a predominant feeling portrayed by the surveyed participants and their relatives. Recognition is something that was repeatedly highlighted as something very positive, together with feelings of security and safety. At the same time, there were participants who did not believe that the GerMoT fulfilled their expectations and that the information about being enrolled was unclear.

This study was, as mentioned, a part of a randomized controlled trial. The inclusion criteria for the main study were based on health care registries without confronting or asking the participants about the current status of their health care. This meant including participants in this study who experienced well-functioning health care. Additionally, there were participants included who had no obvious ongoing health problems despite fulfilling the inclusion criteria. Therefore, it is not surprising that some of these participants and their relatives saw no advantage of being included in the intervention.

The intervention, i.e., the connection to the GerMoT, was given “in addition to the usual care”, as the Swedish health care system provides all citizens with a connection to PC. This connection is reimbursed to the PC by taxpayers and cannot be removed without conflict from the PC and political support. This was not the scope of the GerMoT; thus, the most pragmatic solution was to keep PC and add the GerMoT as a surplus of care. It should also be considered that the GerMoT was a very small entity with only a few employees, which meant that there could be the possibility of someone not being accessible. Therefore, we could not risk that the participants in the intervention group should be without care, aside from acute hospital care; thus, the participants’ connection to PC was continued at the same time as they were enrolled in the GerMoT. This situation, among others, led to uncertainty for both the older people and their relatives and PC about when the “usual care” should be sought and when the GerMoT should be contacted. There were also practical problems in this real-life setting regarding staff turnover and especially a shortage of nurses, which made it difficult to keep everybody informed. Additionally, it took time to find well-working routines and to make everybody involved in the intervention understand what the GerMoT could offer and what it could not. Clarity in communication is formative regarding what expectations a person and their relatives have. More personalized information about the intervention should have been provided, as we all have different care experiences and can perceive the same information in different ways. Therefore, continuous dialogue about how written information is perceived is probably of great importance. Disappointment can mean that the relationship of trust, which is important for such an intervention, dissolves.

Another area mentioned was the time given by the GerMoT to participants who wanted to talk about their many symptoms and worries. In the GerMoT, the patients were given the time they thought they needed, which was in contrast with the PC, where patients felt that the “clock was ticking” because there often was not an opportunity have additional time when needed.

The main findings in this study, i.e., feeling safe and secure and being recognized as an older person with multimorbidity living in the community, is in contrast with other studies that had similar inclusion criteria [13, 14]. The referred to NS was published in 2012; however, subsequent reports do not indicate that the care of older people is improving substantially. For instance, every older person who received care from the municipality saw an average of 16 different carers within a fortnight in 2019 [28], which was an increase in number from 12 in 2007 when this metric was last measured.

Due to many symptoms and progressing illnesses, the presence of tiredness and perhaps cognitive decline make it increasingly difficult for older persons to give a sufficient medical history, which is why continuity of care is of significant importance in terms of giving a feeling of security and safety. In the GerMoT, there was quite a turnover of all professionals—several times—which is why it is depressing that the continuity of care in the GerMoT was mentioned several times as a positive aspect, as this tells us that the turnover of staff in PC is even more frequent. The mentioned reasons why most of the participants found the interventions to be positive could lead to questioning the quality of the “usual care.” This could mean that the conditions in PC should be changed in terms of making the work more attractive to help make physicians and other PC workers want to stay in their positions and to provide better continuity.

If this had not been a part of a randomized controlled study with rigid inclusion criteria, another approach to including participants should have been followed; i.e., there should have been close cooperation between the PC and the GerMoT about which older persons should be given care by the more proactive GerMoT and who should stay in contact with the PC. This approach would also have diminished the risk of unnecessary double work and would have ensured continuous contact between the PC and the older persons when such contact was desired by both parts. Nevertheless, older people are not always able to seek care properly, for instance, due to cognitive decline or other illnesses, or when they are in need of a whole geriatric team, including a specialist in geriatric medicine, for which the GerMoT is a better alternative.

Listening to the older person’s preferences regarding their security and safety experience was in accordance with person-centred care [29–32], which focuses on building a trusting relationship with the person seeking care, listening, and having a holistic view of the person [28]. This description was in accordance with what many participants described regarding being a part of the interventions of the GerMoT but also with what the participants who were in continuous contact with, for example, a specialist geriatric nurse in PC often described.

## Limitation

Some of the older people and relatives who were asked to participate in an interview declined. They did not want to participate because their health status and life situation were extremely strained. If those people who were experiencing markedly poor health at the time of the current study had participated, it might have given us more variation in the data.

Something that could also have affected the older participants’ answers was if their close relatives were present at the interview, which was true on a few occasions. That the relatives refrain from attending was not an option. At the same time, the older people expressed a sense of security that their relatives were present because they had difficulty remembering and even in some cases expressing what they wanted to say themselves, which is when having their relatives present was helpful. Thus, due to the increased feeling of security experienced by older people who had relatives present, the relative’s presence during the interview may have contributed to the interview being carried out. However, there is a risk that these relatives could have influenced the answers given by the older participants. To avoid such an impact, all material that contained an opinion of one of the relatives has been clearly presented as the relative’s opinion in the results.

## Conclusion

This study shows that health care is not adapted to older community dwelling people with multimorbidity who are in need of integrated care. This study offers no evidence regarding exactly how the care of older people with multimorbidity should be organized; instead, it supports the idea that a person-centred approach seems to be more crucial in the care of the person than how the care is organized, i.e., whether it is a special unit such as the GerMoT or a specialist geriatric nurse in PC. The importance of easy accessibility and continuity of care, as recommended by the WHO in 2018 [33], is underlined in the current study. Most likely, a combination of PC, especially with special geriatric nurses, and units such as GerMoT for the most ill and complex older individuals is optimal. The inclusion criteria shown in this and similar studies work well to identify older people who are in need of special attention and around which a discussion should take place between PC and geriatric teams regarding how to plan the best type of care tailored to older individuals. This would allow a feeling of security, safety and a continuity of care even to those with the highest level of health care needs.

## Data Availability

The datasets used and analysed during the current study are available from the corresponding author on reasonable request.

## Abbreviations

Age-FIT: Ambulatory Geriatric-Frailty Intervention Trial
CGA: Comprehensive geriatric assessment
GerMoT: Geriatric Mobile Team
IG: Intervention group
PC: Primary Care
NS: National (Swedish) Survey of older community dwelling people
WHO: Worlds Health Organizarion
